# Research progress on the role of extracellular vesicles in neurodegenerative diseases

**DOI:** 10.1186/s40035-023-00375-9

**Published:** 2023-09-11

**Authors:** Zhengzhe Li, Xiaoling Wang, Xiaoxing Wang, Xiaomei Yi, Yin Kwan Wong, Jiyang Wu, Fangfang Xie, Die Hu, Qi Wang, Jigang Wang, Tianyu Zhong

**Affiliations:** 1 The First School of Clinical Medicine, Gannan Medical University, Ganzhou, 341000 China; 2 Laboratory Medicine, First Affiliated Hospital of Gannan Medical University, Ganzhou, 341000 China; 3 Department of Nephrology, Shenzhen Key Laboratory of Kidney Diseases, and Shenzhen Clinical Research Centre for Geriatrics, Shenzhen People’s Hospital, The First Affiliated Hospital, Southern University of Science and Technology, Shenzhen, 518020 China; 4 State Key Laboratory for Quality Ensurance and Sustainable Use of Dao-di Herbs, Artemisinin Research Center, and Institute of Chinese Materia Medica, China Academy of Chinese Medical Sciences, Beijing, 100700 China; 5 Department of Oncology, The Affiliated Hospital of Southwest Medical University, Luzhou, 646000 China

**Keywords:** Extracellular vesicle, Neurodegenerative disease, Central nervous system, Neurodegeneration, Pathogenesis, Biomarker

## Abstract

Neurodegenerative diseases, such as Alzheimer’s disease, Parkinson’s disease, amyotrophic lateral sclerosis, and Huntington’s disease, affect millions of people worldwide. Tremendous efforts have been put into disease-related research, but few breakthroughs have been made in diagnostic and therapeutic approaches. Extracellular vesicles (EVs) are heterogeneous cell-derived membrane structures that arise from the endosomal system or are directly separated from the plasma membrane. EVs contain many biomolecules, including proteins, nucleic acids, and lipids, which can be transferred between different cells, tissues, or organs, thereby regulating cross-organ communication between cells during normal and pathological processes. Recently, EVs have been shown to participate in various aspects of neurodegenerative diseases. Abnormal secretion and levels of EVs are closely related to the pathogenesis of neurodegenerative diseases and contribute to disease progression. Numerous studies have proposed EVs as therapeutic targets or biomarkers for neurodegenerative diseases. In this review, we summarize and discuss the advanced research progress on EVs in the pathological processes of several neurodegenerative diseases. Moreover, we outline the latest research on the roles of EVs in neurodegenerative diseases and their therapeutic potential for the diseases.

## Introduction

Neurodegenerative disorders such as Alzheimer’s disease (AD), Parkinson’s disease (PD), amyotrophic lateral sclerosis (ALS) and Huntington’s disease (HD) are a heterogeneous group of diseases characterized by gradual progression and selective loss of anatomically or physiologically related neurons, which significantly impair cognitive or behavioral abilities [1–3]. The primary hallmark of neurodegenerative disorders is the accumulation of misfolded proteins into insoluble aggregates (or inclusions) in the central nervous system (CNS), accompanied by progressive neuronal degeneration in the affected regions [4]. The mortality and morbidity associated with these disorders are rapidly increasing owing to the aging of the global population [5].

However, both AD and PD are associated with low detection efficiency due to the lack of available biomarkers [6]. The existing biomarkers allow diagnosis only at advanced stages of disease [7]. Until now, proteomic studies using complete blood, cerebrospinal fluid (CSF), saliva, and urine samples have identified several biomarkers for neurodegenerative diseases [8]. However, the biofluid-based methods have limitations, such as the extremely low concentrations of the protein biomarkers (estimated to account for less than one-millionth of the total CSF proteins and one ten-billionth of the total blood proteins) [9]. The blood–brain barrier (BBB) prevents the free passage of molecules between the CNS and blood, leading to a difference between CSF and blood. However, CSF collection is invasive in nature and is unacceptable when early symptoms of disease are not apparent [10]. In addition, the biomarkers are also expressed in other tissues in addition to the brain, which may confound their measurement in biofluids. Currently, the gold standard for the diagnosis of neurodegenerative diseases remains brain imaging, either magnetic resonance imaging or positron emission tomography [11]. Although imaging is highly sensitive, its accuracy depends on the experience and skill of the operator [12]. Given these clinical challenges, the discovery of specific in vivo biomarkers, including biofluid and molecular imaging biomarkers, is a major research priority for neurodegenerative disorders.

Extracellular vesicles (EVs) are essential mediators of communication between cells. In the CNS, EVs transmit signaling information between nerve cells and contribute to their development and function [13]. The EVs released from the brain and the spinal cord are proposed to be unique to the CNS [14]. In neurodegenerative disorders, pathological molecules are transferred to healthy tissues by EVs to perform pathological functions. Additionally, EVs are thought to play a protective role by expelling pathological molecules from cells [15]. In this review, we outline the evidence for the interaction of EVs with many of the specific proteins, nucleic acids and lipids implicated in neurodegenerative diseases, demonstrating that EVs are key regulators of neuronal dysfunction and death and play a central role in cell-to-cell communication and neurodegenerative disease progression. In addition, we discuss the latest research on the therapeutic potential of EVs for neurodegenerative diseases.

## Classification of EVs

**Fig. 1:**
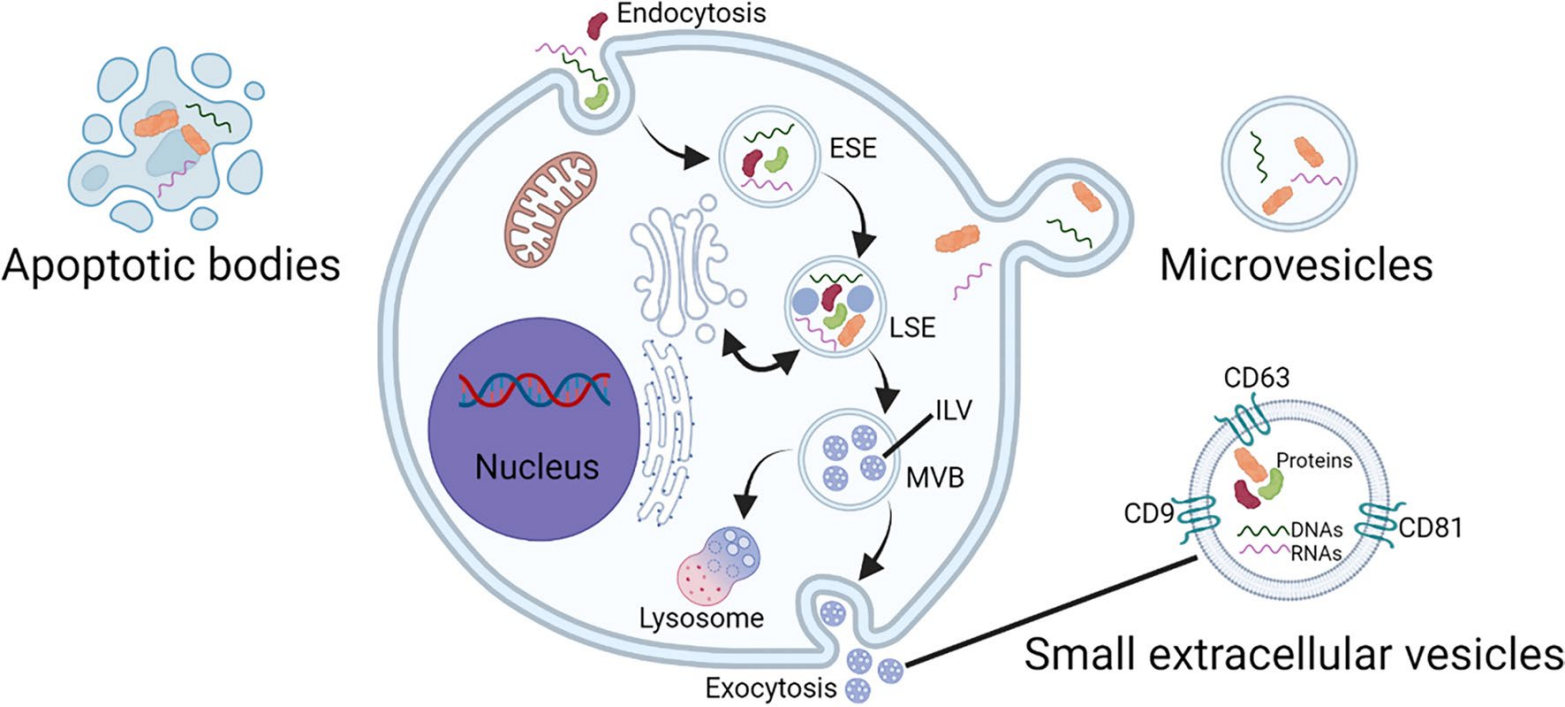
Classification and biogenesis of extracellular vesicles. Cells can assimilate extracellular substances by plasma membrane invagination and endocytosis. The late sorting endosomes (LSEs) are transformed from vesicles fused with the early sorting endosomes (ESEs). Intraluminal vesicles (ILVs) are caused by a second invagination of the LSEs. Multivesicular bodies (MVBs), further transformed from LSEs, can fuse with lysosomes or autophagosomes for degradation, or with the plasma membrane to release ILVs, which are termed small extracellular vesicles. Microvesicles are produced from the outward budding and fission of the plasma membrane. Apoptotic bodies are large vesicles formed by apoptotic cells

EVs are nanosized vesicles (30–2000 nm) with lipid bilayer membranes, which are actively secreted by almost all cells. The membrane structure of EVs protect the contents from destruction by the extracellular environment [16, 17]. EVs can be divided into small extracellular vesicles (sEVs), microvesicles (MVs), and apoptotic bodies based on their dimensions, as proposed by the MISEV guidelines [18] (Fig. 1).

sEVs (40–200 nm in diameter) are smallest EVs and are secreted by various living cells [19]. Fundamentally, sEVs are generated within cells through the endosomal pathway in three stages. First, the plasma membrane is invaginated to produce endocytic vesicles, some of which fuse to form early sorting endosomes (ESEs); this process involves the participation of proteins such as endosomal sorting complex required for transport (ESCRT) proteins, tetraspanin proteins (CD9, CD63, and CD81), apoptosis-linked gene 2-interacting protein X (Alix), and tumor susceptibility gene 101 (TSG101). Subsequently, these ESEs may exchange materials with other organelles or fuse with different ESEs to transform into late sorting endosomes (LSEs), which can further transform into multivesicular bodies (MVBs). MVBs generate many intraluminal vesicles (ILVs) to pack intracellular substances; this process involves RAB GTPase proteins and cytoskeletal proteins such as actin and tubulin. Next, the MVBs fuse with the plasma membrane and release ILVs into the extracellular space, where they are defined as sEVs; the secretion of sEVs requires the participation of the SNARE protein complex and the synaptotagmin family [20–22].

MVs (100–1000 nm) were previously called “the platelet dust” and are generated through outward budding and fission of the plasma membrane; therefore, the membrane composition of MVs is close to that of the donor cells [23]. The production of MVs is related to the asymmetric distribution of phospholipids in the cell membrane bilayer [24]. Calcium influx can disrupt the asymmetry of phospholipids by activating phospholipid scramblase to redistribute phospholipids in the cell membrane bilayer. Simultaneously, calcium-dependent proteolytic enzymes degrade the membrane-bound cytoskeleton and initiate the production of MVs [25]. Some researchers have shown that ARRDC1 (arrestin-domain-containing protein 1) can recruit the ESCRT proteins TSG101 and Vps4 to the cell membrane to initiate membrane budding [26].

Apoptotic bodies (500–2000 nm) are comparatively giant vesicles derived from apoptotic cells, and contain cytoplasm, organelles, and nuclear debris [23]. Blebbing from the plasma membrane during apoptosis leads to formation of apoptotic bodies in the form of MVs [27].

## EV cargos

**Fig. 2:**
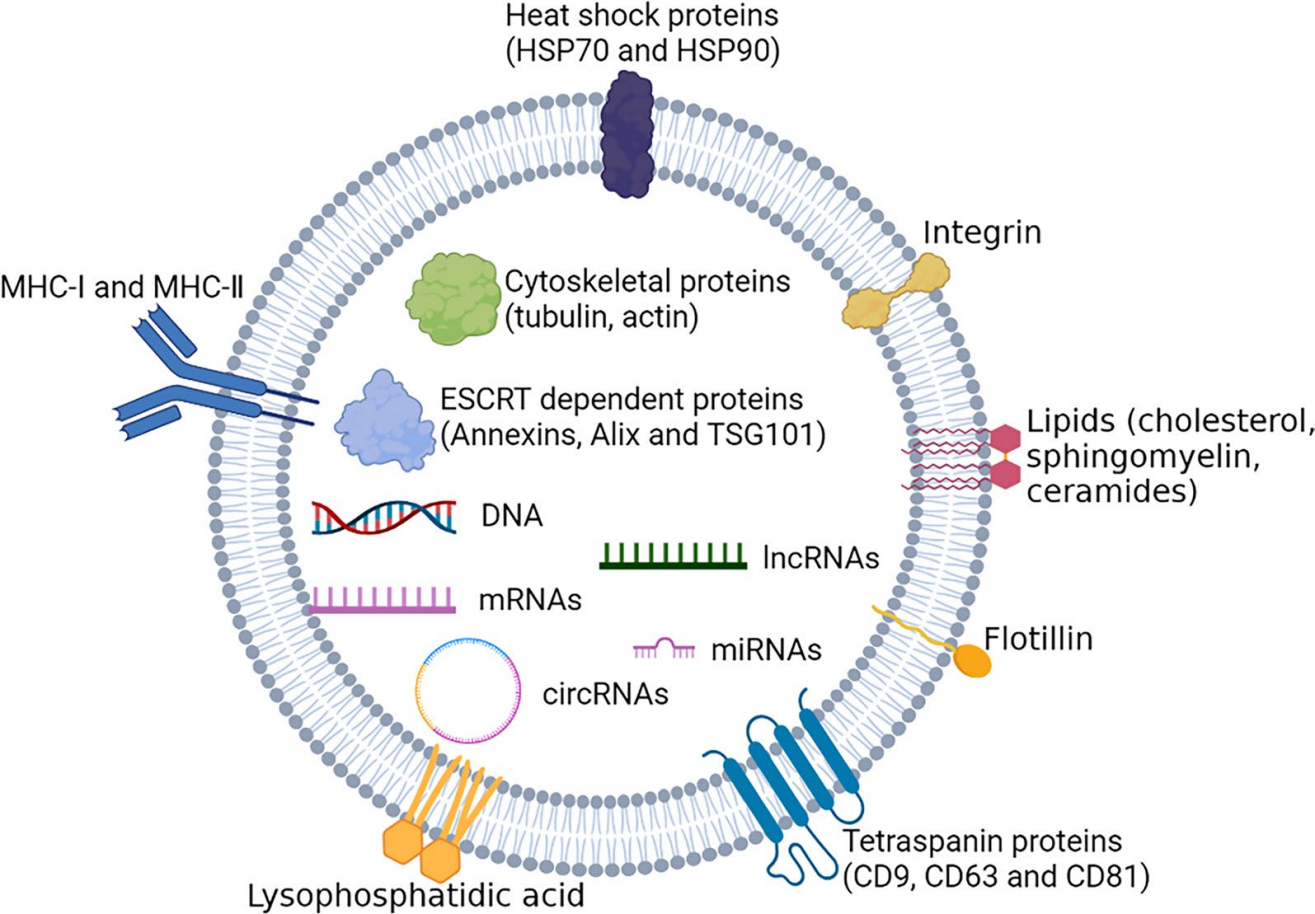
Structure of extracellular vesicle. The phospholipid bilayer encapsulates different types of membrane proteins, intracellular proteins, DNA, RNA, lipids, and metabolites to form EVs. Several membrane and intracellular proteins are used as EV markers, including TSG101, Alix, CD63, CD9, and CD81

The application of mass spectrometry and high-throughput sequencing has enabled large-scale screening and characterization of EV contents [28]. EVs contain various bioactive molecules, including soluble proteins, nucleic acids, lipids and metabolites, all of which play crucial roles in cell-to-cell communication and are responsible for delivering various signaling molecules to both proximal and distant locations (Fig. 2) [29].

The proteins in EVs are mainly divided into two types: (1) ubiquitous proteins, such as those participating in the formation of EV structure, including cytoskeleton components (tubulin, actin, and microfilament-associated protein); certain conserved proteins, including those involved in ESCRT-dependent biogenesis (Annexins, Alix, and TSG101); ESCRT-independent tetraspanin family proteins, such as CD9, CD63, and CD81; and heat shock proteins, such as HSP70 and HSP90 [30]; and (2) proteins related to the original cell. For example, EVs derived from antigen-presenting cells are abundant in major histocompatibility complex (MHC)-I (MHC-I), MHC-II, CD80 and CD86; platelet-derived EVs contain the factors of hemophilia and integrin CD41A; and proteins abundant in the EVs of tumor cells, such as Fas ligand and transforming growth factor-β, are frequently related to tumorigenesis [31]. These proteins, which are absent on other types of vesicles, can be considered “markers of EVs” [32].

Nucleic acid fragments in EVs are usually ~ 200 bp in length, and some of them can be translated into functional proteins that affect the biological function of the recipient cell [33]. EVs contain many nucleic acids, including genomic DNA [34] and mitochondrial DNA [35], as well as RNA (mRNA, microRNA [miRNA], lncRNA, and circRNA) [36, 37]. Considered as the primary regulator of recipient cell activity, RNA can regulate the gene expression and function of target cells by directly participating in transcription, post-transcriptional processing, as well as protein translation and modification [38], and play a regulatory role in the biological function of cells and the progression of diseases [39]. miRNAs are small, 22-nt-long, non-protein-coding RNAs that induce posttranscriptional gene silencing by binding to their complementary mRNA targets and inhibiting translation and/or inducing degradation of mRNA [40]. Under physiological conditions, miRNA-dependent gene regulation ensures precise protein output and minimal protein expression noise [41]. miRNAs in the CNS control gene expression in various cell types in a highly regulated temporospatial pattern and a neuronal activity-dependent manner [42]. In addition, the remarkable stability of miRNAs in the extracellular environment and hence in body fluids, together with the availability of sensitive methods for their detection and quantitation, has led to the wide use of circulating miRNAs as biomarkers for various human disorders [43]. Meanwhile, the miRNA-based therapeutics mainly comprise synthetic miRNAs to restore endogenous miRNA levels (e.g., miRNA mimics) or antisense inhibitor oligonucleotides to reduce functionally available endogenous miRNAs, such as antamiRNAs and antagomiRNAs.

EVs are generally enriched with lipids, including cholesterol, sphingomyelin, ceramides, sphingolipids, glycerophospholipids, and glycosphingolipids [44]. Lipids including phosphatidylcholine, phosphatidylserines and phosphatidylinositols are found in lower quantities [45]. The lipid composition of EVs generally represents that of the donor cell. The membranes of EVs contain lysophosphatidic acid, which is central to the formation of ILVs from multivesicular endosomes [46]. These lipids not only participate in the biosynthesis and uptake of EVs, but also act as a class of bioactive molecules in various biological processes, including immunological surveillance, modification of the tumor microenvironment, and regulation of inflammation [47, 48]. The lipids in EVs may also be used as biomarkers for disease diagnosis and treatment [49].

## Functions of EVs in the CNS

A common feature of neurodegenerative diseases is the misfolding, aggregation and accumulation of pathological amyloids inside or outside the brain cells. Accordingly, detection of these pathological proteins in body fluids and tissues may be a powerful tool for early diagnosis of these diseases. Effective therapies to delay or prevent the onset and progression of neurodegenerative diseases have not been established to date. In the past decade, EVs have been reported as novel and important carriers of signaling molecules in vivo. A growing body of literature has highlighted an important role of EVs in the cell-to-cell transmission of pathogenic protein aggregates, thereby contributing to the pathological and clinical progression of neurodegenerative diseases [50]. These EVs can carry and protect a range of proteins, lipids, and nucleic acids from degradation in the extracellular space [51]. After being delivered to target cells, EVs can influence the physiology of the recipient cells [52]. It has been observed that EVs are released by neurons, oligodendrocytes, microglia, and astrocytes in the CNS [53]. Recently, several studies have revealed the physiological roles of EVs in the CNS, including regulation of glutamatergic synaptic activity during nerve cell development. Astrocytes maintain brain homeostasis by internalizing miR-124 from microglia-derived EVs to regulate levels of glutamate transporter 1 and glutamate uptake [54]. Non-neuronal cells promote neurite growth and neuron survival through the EV-mediated release of neuroactive substances such as Hsp70 and synaptophysin I [55, 56]. Another study showed that stimulation of serotonin receptors increases the release of insulin-degrading enzymes from microglia via EVs, which are capable of degrading the neurotoxic peptide amyloid β (Aβ) [57]. EVs from human bone marrow-derived endothelial progenitor cells are able to repair the damaged microvasculature in the CNS of symptomatic SOD1-G93A mutant mice [58]. Additionally, some EVs play a key role in the pathological processes of neurological diseases. Sardar Sinha et al*.* [59] found that the EVs from AD brains contain abundant toxic Aβ and promote the progression of AD by spreading Aβ between neurons. Moreover, Guo et al*.* [60] found that microglial exosomes promote the intercellular transmission of α-synuclein (α-syn), and induce neurodegeneration in the substantia nigra and striatum, a key mechanism of PD pathogenesis. Therefore, EVs not only play an important role in CNS development, neuroprotection, repair, and further regulation of neuronal activity, but are also involved in the occurrence and development of CNS diseases.

As EVs can cross the BBB into the blood, neurally derived EVs are present in both the bloodstream and CSF [61]. Although EVs can be isolated from CSF, plasma, and serum, the neurally derived EVs are more concentrated in the CSF and have more specific diagnostic and research value for neurological diseases. However, isolation of EVs from CSF is currently not very feasible due to the complicated and difficult process of CSF collection. Therefore, detection of EVs in human blood (plasma and serum) is a relatively simple and powerful approach, although it may be complicated by the presence of admixtures from multiple sources in blood, including serum proteins or a mixture of EVs from other organs [62]. In general, detection of EVs from both CSF and blood each have their advantages, and the roles of these EVs in the pathology and diagnosis of neurodegenerative disorders are summarized below.

### EVs and AD

AD is clinically characterized by progressive cognitive decline, and pathologically by plaques comprising Aβ peptide and nerve fiber tangles containing the hyperphosphorylated Tau protein [63, 64]. The Aβ peptide is produced from cleavage of amyloid precursor protein (APP), and the excessive phosphorylation of tau protein can lead to the separation of tau from microtubules and mutual aggregation, leading to formation of neurofibrillary tangles and deposition in axons and dendrites [65]. About 10% of AD cases occur in an early-onset autosomal dominant manner; they are called familial cases. The following three proteins are associated with familial AD cases: APP, which is sequentially cleaved by β and γ secretases to produce Aβ, and presenilins 1 and 2 (PS1 and PS2) which are subunits of the γ-secretase [66]. A recent study showed that the protease-containing plasma EVs may be part of the communication axis between the brain and the periphery, and they accelerate the pathogenesis of AD in transgenic mouse models by splitting APP or other substrates in target neurons, providing evidence for the pathogenic role of plasma EVs in AD [67, 68]. Therefore, the transmission of EVs between cells is an important factor in the pathogenesis and development of AD (Table 1).

**Table 1:** The roles of extracellular vesicles in Alzheimer’s disease

Source of EVs	Content with biomarker potential	Mouse and cell models for mechanistic studies	Downstream molecules or pathways	References
Brain tissue, CSF, and blood	Aβ	AF22 cells, SH-SY5Y cells and 5XFAD mice	NA	[59, 69–72]
CSF and blood	Tau	N2a cells, neurons, and App^NL−G−F^ mice	NA	[73–76]
Brain tissue, CSF, and plasma	Aβ_1-42_, Aβ oligomer, p-tau 181, and p-tau 396	NA	NA	[77–79]
CSF	miR-16-5p, miR-451a, miR-605-5p, and miR-125b-5p	NA	MAPK signaling pathway	[80]
CSF	miR-135a	HT-22 cells, neurons, and APP/PS1 mice	NA	[81]
Plasma	Aβ_42/40_ and miR-384	NA	NA	[82]
Plasma	miR-29c-3p	NA	NA	[83]
Plasma	let-7g-5p, miR126-3p, miR142-3p, miR-146a-5p, and miR-223-3p	NA	p53, toll-like receptor signaling pathway, MAPK signaling pathway, NF-kappa B signaling pathway, Alzheimer’s disease pathway, apoptosis, PI3K-Akt signaling pathway, cell cycle and cytokine-cytokine receptor interaction	[84]
Serum	miR-193b	HT-22 cells and APP/PS1 mice	NA	[85]
Serum	miR-135a, miR-193b, and miR-384	NA	NA	[86]
Serum	miR-125b and miR-361	NA	NA	[87]
Serum	miR-30b-5p, miR-22-3p, and miR-378a-3p	NA	Proteoglycans in cancer, viral carcinogenesis, signaling pathways regulating pluripotency of stem cells, and cellular senescence	[88]
Plasma	miR-23a-3p, miR-223-3p, miR-190a-5p, and miR-100-3p	Neurons	Axon guidance and long-term depression	[89]
Plasma	let-7e	Neurons, microglia	IL-6	[90]
BM-MSCs	miR-146a	APP/PS1 mice	IRAK1, TRAF6, and NF-κB	[91]

*NA* No accessible data in the study; *CSF* Cerebrospinal fluid; *N2a cells* Mouse neuroblastoma Neuro-2a cells, *HT-22 cells* Hippocampal neuronal cell line, *BM-MSCs* Bone marrow mesenchymal stem cells

Growing evidence has shown that small EVs (for example, exosomes) can serve as biomarkers for neurodegenerative diseases and that they are more reliable than conventional specimens such as pure CSF, blood and urine [92]. Initial evidence for a link between EVs and AD came from a study showing the accumulation of EV proteins such as flotillin-1 and Alix around amyloid plaques in the brains of AD patients, and Aβ peptide release in association with EVs [93]. Recently, Aβ has been shown to be stored either in the lumen or on the surface of microglial EVs [94]. More importantly, Joshi et al. reported in 2014 the first evidence for the neurotoxicity of EVs carrying Aβ[95]. Later, several in vivo studies provided further evidence that Aβ in association with EVs is associated with AD. The CSF EVs of patients with AD contain high levels of Aβ, which can cause damage to neurons in vitro and in animal models in vivo [59, 96]. A recent study has indicated that large microglial EVs containing Aβ are capable of escalating and propagating early synaptic dysfunction in AD between entorhinal cortex and dentate gyrus in the mouse brain by moving at the surface of neurons [69]. Additionally, the Aβ levels in neurally derived EVs in plasma or serum are gradually elevated during disease progression in AD patients compared to asymptomatic individuals, and can thus be used as a biomarker for disease diagnosis and to define disease progression [70]. It has been proposed that the EVs may stimulate Aβ aggregation and interfere with the uptake of Aβ aggregates by astrocytes and microglia, leading to Aβ aggregation in AD [71, 73].

The plasma or serum EVs of AD patients also contain tau protein, which can be transferred to neurons, causing tau accumulation in neurons — another major feature of AD [70, 72]. Additionally, total tau (t-tau) and hyperphosphorylated tau (p-tau) have been detected in the EVs of primary neuron cultures and neuroblastoma cells overexpressing tau [97]. In neurodegenerative diseases, activated microglia release higher levels of EVs than inactivated microglia, and in AD, these activated microglia secrete EVs containing tau protein, which can propagate to neurons and promote the progression of AD [74, 98]. However, the rapid tau propagation from the entorhinal cortex to the dentate gyrus can be dramatically suppressed by depletion of microglia and inhibition of EV synthesis [99]. The EVs secreted by induced pluripotent stem cells carrying mutant A246E PS1 contain high levels of APP and induce tau deposition in mouse brains after in vivo injection [75]. A recent study compared the diagnostic capabilities of neuronal-derived plasma EV and CSF Aβ1-42, p-tau181, and t-tau and found that the combination of these biomarkers in either EVs or CSF had superior diagnostic performance than each single biomarker [76]. Meanwhile, other studies have shown that the levels of soluble Aβ1-42, Aβ oligomer, p-tau181, and p-tau396 in EVs isolated from brain tissue, blood, and CSF of AD patients are significantly increased before the clinical diagnosis of AD, suggesting that these biomarkers from EVs can be used for AD diagnosis [77, 78]. The above studies indicate that EVs are closely related to the pathological process of AD. Although EVs are involved in AD, their role in the pathological process remains controversial. Aβ and p-tau proteins are neurotoxic proteins that can cause AD, and EVs have been shown to promote their tansfer and diffusion [79]. However, other studies have shown that microglia can improve the pathological phenotype of AD by uptaking Aβ through EVs and transforming it into neuroprotective substances [100].

In addition to proteins, many miRNAs involved in AD progression have been found in AD-derived EVs and can also serve as biomarkers. miRNA profiles in the brains of AD patients are altered compared to healthy controls, often in a stage- and/or region-specific manner. How these alterations impact disease onset and progression and whether they act as a cause or an effect along the disease trajectory remains unclear. Nevertheless, the specific early miRNA aberrations in human brains indicate that disruption of miRNA homeostasis may act as a (co-)driver of certain pathological cascades [101].

In AD, changes in EV miRNAs that target APP processing, tau phosphorylation, and mitochondrial- and apoptosis-related genes that regulate neurodegenerative events in AD, have received much attention [102]. A recent study has shown that SNORDs–a group of Box C/D small nucleolar RNAs – are enriched differently in EVs isolated from the plasma of AD patients compared to controls [103]. McKeever et al*.* [80] demonstrated that EVs containing miR-16-5p, miR-451a, and miR-605-5p are decreased, and those containing miR-125b-5p are increased in the CSF of patients with early-onset AD compared to healthy controls. Analysis of mRNA targets of miR-16-5p, miR-451a, miR-125b-5p, and miR-605-5p revealed that these miRNAs are related to neuronal projection, synaptic signaling, metabolism, apoptosis, and the immune system. By comparing EVs miR-135a in CSF vs serum, Liu et al*.* [81] reported that the increased level of miR-135a in ABCA1-labeled EVs in CSF is more effective for the early diagnosis of AD. However, compared to CSF, EVs in peripheral blood are easier to obtain and detect. Li et al*.* [82] reported that Aβ42/40 and miR-384 in NCAM/ABCA1 dual-labeled plasma EVs have potential advantages in diagnosing subjective cognitive decline (SCD), i.e., the early stage of AD. The EVs with dual‐specific biomarkers can be obtained through a combination of magnetic bead method and the microtiter plate method, and used to achieve ideal AD diagnostic performance. This provides a new direction for future EV research, although these findings need to be further confirmed in future studies. Another study using the same approach showed a potential advantage of miR-29c-3p in NCAM/amphiphysin 1 dual-labeled EVs in plasma in the diagnosis of SCD [83]. It has been shown that double-labeled EVs from plasma have promising applications in the diagnosis of AD, and could potentially serve as a substitute for CSF markers. Aharon et al*.* [84] found that the let-7 g-5p, miR126-3p, miR142-3p, miR-146a-5p, and miR-223-3p levels in plasma EVs are correlated with disease severity and could be used as biomarkers to reflect the severity of AD. One study suggested that miR-193b in ABCA1-labeled serum EVs contributes to the early diagnosis of AD [85], although the use of ABCA1-labeled EVs from serum for AD diagnosis needs to be confirmed in future studies.

For most diseases, combined biomarkers are likely to have better diagnostic performance than a single one. Yang et al*.* [86] analyzed miR-135a, miR-193b, and miR-384 in serum EVs and demonstrated that the combination of miR-135a, miR-193b, and miR-384 had an outstanding diagnostic performance with an area under the curve (AUC) value of 0.997 and can be used for early diagnosis of AD. They found that miR-135a and miR-384 were upregulated and miR-193b was downregulated in the serum EVs of patients with AD. Another study showed that the combination of miR-125b and miR-361 in serum EVs had a high diagnostic efficacy, with a sensitivity of 91.67%, selectivity of 95.00%, and accuracy of 99.52% [87]. Moreover, a combination of miR-30b-5p, miR-22-3p and miR-378a-3p in serum EVs has good diagnostic capabilities, with AUC of 0.880 [88]. These data suggest that the combination of miRNAs from EVs in peripheral blood has potentials to distinguish AD from healthy controls. Nakano et al*.* [91] showed that the level of miR-146a was increased in the hippocampus of APP/PS1 AD model mice injected with bone marrow mesenchymal stem cells (BM-MSCs), and this upregulation was caused by the secretion of exosomal miR-146a from BM-MSCs and involved in the correction of cognitive impairment. Through a method of isolating neuron-derived EVs from plasma based on neuronal expression of L1 cell adhesion molecule (L1CAM), one study showed that the levels of miR-23a-3p, miR-223-3p, and miR-190a-5p in neuron-derived EVs isolated from the plasma of AD patients were significantly increased, whereas the level of miR-100-3p was significantly decreased [89]. Another study showed that the neuron-derived EVs in AD patients induce neuroinflammatory responses in microglia, and that the neuron-derived EV let-7e is a potential biomarker for AD [90]. However, a recent study reported that L1CAM may not be a good marker for neuron-derived EVs, as L1CAM is not associated with EVs in human CSF or plasma, disputing the use of this marker to isolate neuron-derived EVs [104]. To overcome these limitations, there is an urgent need to develop better separation methods or alternative markers.

In conclusion, we have noticed that numerous potential biomarkers have been identified by bioinformatics analyses and tested in experimental studies of EV miRNAs. However, similar to the current status of research on protein contents of EV, these studies were focused on the discovery of new biomarkers without clinical validation. There is a lack of consensus among research groups, and more practical work is needed for translation to clinical application.

### EVs and PD

PD is clinically characterized by progressive rigidity, bradykinesia, and tremor [105]. Significant pathological changes associated with PD include the degenerative death of dopaminergic (DA) neurons in the substantia nigra, leading to a significant reduction of DA in the striatum and the presence of Lewy bodies in residual nigrostriatal neurons [106]. The aggregated α-syn plays a role in neurodegeneration, and the latter is a cause of the symptoms associated with PD. EVs have received much interest as a potential player in PD and in vitro studies have shown the α-syn-carrying potential of EVs since as early as 2010, paving the way for the extracellular seeding theory. Other studies have shown that the CSF EVs from PD patients can cause α-syn aggregates in target cells and could lead to the disease pathology [107].

The correlation between EVs and PD was first confirmed by the in vitro transmission of EVs and in vivo experiments of misfolded toxic proteins in PD (Table 2). Lee et al*.* [108] confirmed that both primary cortical neurons of rats and RA (all-trans-retinoic acid)-differentiated SH-SY5Y neurons can secrete EVs containing α-syn. These results suggest that vesicle-mediated release of the monomeric and oligomeric forms of α-syn contributes to proteasome defects and mitochondrial dysfunction in the pathogenesis of PD. The propagation of α-syn through EVs along multiple brain regions represents one of the central mechanisms of PD progression. Other studies have confirmed that lysosomal dysfunction may be one of the main factors accelerating PD pathology, as the release of EVs containing α-syn is increased when intracellular protein transport through lysosomes is blocked [109, 110]. Emerging evidence suggests that EVs play a role in the intercellular diffusion of aggregating α-syn, resulting in prion-like diffusion of the aggregates [111]. Neuroblastoma cells express α-syn protein in EVs and release it into the medium for extracellular transport [112]. These observations suggest that although α-syn may be released independently of EVs, it can also be released via EVs.

**Table 2:** The role of extracellular vesicles in Parkinson’s disease

Source of EVs	Content with biomarker potential	Mouse and cell models for mechanistic studies	Downstream pathways or molecules	References
N2a cells, microglia	α-syn	NA	NA	[60, 111–114]
Plasma	PrP	NA	NA	[115]
CSF	Let-7f-5p, miR-125a-5p, miR-27a-3p, miR-423-5p, and miR-151a-3p	NA	SNCA	[116]
CSF	miR-1, miR-19b-3p, miR-153, and miR-409-3p, miR-10a-5p, and let-7g-3p	NA	Dopaminergic synapse and cholinergic synapse	[117]
Serum	miR-21-3p, miR-22-3p, miR-223-5p, miR-425-5p, miR-21-3p, and miR-199a	NA	Fatty acid biosynthesis, ECM-receptor interaction, fatty acid metabolism, and hippo signaling pathway	[118]
Serum	miR-374a-5p, miR-374b-5p, miR-199a-3p, miR-28-5p, miR-22-5p, and miR-151a-5p	NA	NA	[119]
Serum	let-7d, miR-22*, miR-23a, miR-24, miR-142-3p, and miR-222	NA	NA	[120]
Serum	miR-24, miR-195, and miR-19b	NA	NA	[121]
Serum	miR-29c	NA	NA	[122]
Plasma	miR-331-5p and miR-505	NA	NA	[123]
Plasma	let-7e-5p	NA	TLR7	[124]
Plasma	miR-15b-5p, miR-30c-2-3p, miR-138-5p, miR-106b-3p, miR-338-3p, and miR-431-5p	SH-SY5Y cells	Dopaminergic synapse and Parkinson's disease pathways	[125]

*NA* No accessible data in the study; *CSF* Cerebrospinal fluid; *N2a cells* Mouse neuroblastoma neuro-2a cells; *PrP* prion protein

Other evidence also supports this hypothesis, including the fact that lysosomal dysfunction (a PD-related stress state) increases α-syn release through EVs. Emmanouilidou et al. reported that impairment of lysosomal acidification increased the levels of secreted α-syn. In addition, BFA, an effective inhibitor of the classical ER/Golgi-dependent pathway that induces disruption of the classical secretory pathway, did not alter the levels of secreted α-syn; in contrast, α-syn was released by externalized vesicles in a calcium-dependent manner, suggesting a non-classic secretory pathway for α-syn [126]. Additionally, the presence of EVs has been shown to increase the tendency of α-syn aggregation, and cultured cells have been found to be more likely to absorb EV-associated α-syn than free α-syn oligomers, suggesting that α-syn is transferred between cells via EVs [113]. Grey et al*.* [111] further demonstrated that pro-inflammatory cytokines, such as TNF-α, IL-1β and IL-6, increase the aggregation of α-syn in neuron-derived EVs. Additionally, direct injection of EVs from α-syn-treated microglia into the mouse striatum resulted in α-syn phosphorylation and aggregation, degeneration of DA neurons in several brain regions associated with the striatum, and dyskinesia. It has also been found that the α-syn oligomer in the microglia-derived EVs in the CSF of PD patients could induce α-syn aggregation in neurons [60]. In addition to α-syn diffusion, EVs have also been implicated in the transfer of mutated leucine-rich repeat kinase 2 (LRRK2) acting as a risk factor for developing PD [127].

To date, no reliable, clinically applicable biomarkers have been established for PD. One of the pathological hallmarks of PD is the presence of Lewy bodies in surviving neurons, which consist of insoluble aggregated proteins, with α-syn being the major component [128]. However, α-syn is easily secreted into the extracellular spaces and has been identified in CSF, blood, and saliva [129]. Although the mechanisms of α-syn secretion are not fully understood, Shi et al. have demonstrated that the diagnostic sensitivity and specificity of plasma exosomal α-syn are comparable to those of CSF α-syn [130]. Another study showed that the concentration of α-syn in plasma EVs may serve as a potential diagnostic biomarker for PD [114]. Furthermore, Jiang et al. showed that the combined serum neuronal exosome-associated α-syn and clusterin outperform any previously reported blood-based assay or CSF total or pathogenic α-syn in predicting PD from atypical parkinsonism in clinical and prodromal PD [131], while studies by Ohmichi et al. demonstrated the quantification of brain-derived EVs in plasma as a biomarker to diagnose PD [115].

In addition, proteins are not the only bioactive content of EVs studied in the context of PD. miRNAs, through their epigenetic control of recipient cells, have shown great impacts on the pathological mechanisms of numerous diseases. Previous studies have found that circulating miRNAs are closely related to the pathophysiological processes of PD and can be easily collected using non-invasive or minimally invasive techniques, making them promising biomarker candidates for PD [132]. miRNAs are highly stable and resistant to degradation in EVs, and recent studies have shown that EV miRNAs play an important role in both physiological and pathological status of PD and can be used as biomarkers of PD [133]. Dos Santos et al*.* [116] analyzed EV miRNAs from CSF samples of 40 early PD and 40 healthy controls in a cross-sectional cohort, and conducted small RNA sequencing, protein-binding ligand assays, and machine learning. The results showed that the expression levels of let-7f-5p and miR-125a-5p were increased, while those of miR-27a-3p, miR-423-5p and miR-151a-3p were decreased. The combination of miR-22-3p, miR-10b-5p, miR-151a-3p and α-syn had the best diagnostic performance for PD with a sensitivity of 97%, specificity of 90%, and AUC of 96%. Gui et al*.* [117] found that the expression of miR-1 and miR-19b-3p was significantly decreased, while the expression of miR-153, miR-409-3p, miR-10a-5p and let-7 g-3p was significantly increased in CSF EVs of PD patients. Manna et al*.* [118] reported that a set of miR-21-3p, miR-22-3p and miR-223-5p in serum EVs can discriminate PD from healthy controls with an AUC of 0.75. Additionally, it was found that the combination of miR-425-5p, miR-21-3p and miR-199a in serum EVs had a good performance in discriminating between progressive supranuclear palsy and PD, with an AUC of 0.86. He et al*.* [119] reported that six serum-derived EV miRNAs, including miR-374a-5p, miR-374b-5p, miR-199a-3p, miR-28-5p, miR-22-5p and miR-151a-5p, may be used as biomarkers for early diagnosis and progression of PD. The biological functions of these miRNAs in the occurrence and development of PD need to be further studied. The expression levels of let-7d, miR-22* (asterisk indicates anti-sense miR), miR-23a, miR-24, miR-142-3p and miR-222 were found to be significantly increased in serum EVs of PD patients, which can improve clinical diagnosis of PD [120]. A comparison of 24 miRNAs in serum EVs between 109 patients with PD and healthy controls showed that the levels of miR-24 (AUC 0.908) and miR-195 (AUC 0.697) were increased, whereas miR-19b (AUC 0.753) was decreased in PD. Therefore, they may represent novel biomarkers [121]. Ozdilek et al*.* [122] compared the expression levels of miR-19a, miR-19b, miR-29a, miR-29c, miR-181, miR-195 and miR-221 in serum EVs between 51 PD patients and 20 healthy controls. The results showed that the expression level of miR-29c was significantly increased in PD with an AUC of 0.689. In another study, RT-qPCR results showed that the expression level of miR-331-5p in plasma EVs of PD patients was significantly increased, while that of miR-505 was significantly decreased, with AUCs of 0.849 and 0.898, respectively, suggesting that they have potential value for early diagnosis of PD [123]. In another study, Nie et al*.* [124] pointed out that in plasma EVs from 34 normal controls, 5 donors with AD and 7 donors with PD, miR-125a-5p, miR-1468-5p, miR-204-5p, let-7e-5p, miR-375, miR-369-5p, miR-423-5p and miR-23a-3p were significantly increased/decreased. Among them, let-7e-5p expression was increased in patients with PD. Besides, they found that the level and quality of miRNAs in EVs were better than those in plasma, suggesting that biomarkers in plasma EVs have better diagnostic efficiency. Xie et al*.* [125] suggested that miR-15b-5p, miR-30c-2-3p, miR-138-5p, miR-106b-3p, miR-338-3p and miR-431-5p in plasma EVs represent potential biomarkers for PD diagnosis. Studies on SH-SY5Y cells treated by MPP^+^ demonstrated that the target genes of these miRNAs may be enriched in KEGG dopaminergic synapse pathway and PD pathway. It is not difficult to see that due to the influence of other components in blood, the diagnostic performance of blood EVs is weaker than that of CSF. Efforts should be made to develop a method to perfectly mark neurally derived EVs in blood.

Taken together, these observations provide a proof-of-concept that modulating miRNA levels in PD brains may concomitantly modify multiple aspects of PD pathology, and miRNAs may be candidate targets for intervention as common downstream regulators of functionally diverse molecular pathways in PD. Although some of these miRNAs have been previously studied and discussed in this paper, experimental testing of newly identified miRNA correlations in appropriate model systems is critical for drug development.

###  EVs and ALS

ALS causes weakness and atrophy of the muscles of the extremities, trunk, and chest following motor neuron injury. The clinical manifestations of ALS are progressive muscle weakness, atrophy, and spasticity, reflecting the degeneration of upper and lower motor neurons in the cortex, brainstem, and spinal cord. The pathogenesis of ALS includes an imbalance of protein homeostasis in the nervous system, prion-like proliferation and reproduction of abnormal proteins, mitochondrial dysfunction, and inflammatory cascade responses. Approximately 90% of cases are sporadic and 10% are familial. Furthermore, about 20% of familial cases are caused by mutations in Cu/zinc superoxide dismutase (*SOD*) [134]. Mutations in the *SOD1* gene lead to abnormal folding of SOD1 protein in vivo, ultimately leading to the formation of toxic aggregates [135]. Multiple ALS-related mutations have also been found in the Tar DNA binding protein-43 (*TDP-43*) gene [136]. TDP-43, a member of the heterogeneous nuclear ribonucleoprotein (hnRNP) family, is involved in RNA processing and can form insoluble aggregates in the brains of patients with ALS [137].

Over the years, many molecular targets have been suggested to be involved in the pathogenesis of ALS, and several proteins encoded by genes involved in the pathogenesis of ALS have been identified. Recent evidence suggests that many of these proteins are present or differentially expressed in EVs and spread between neurons and glial cells within different brain regions, contributing to their transmission and propagation (Table 3). These proteins include SOD1 [138], TDP-43 [139], Fused in sarcoma (FUS) [140], and Dipeptide repeating proteins (DPRs) [141]. The first ALS-related gene mutation was found in *SOD1*. SOD1 was first detected in EVs derived from murine motor neuron-like NSC-34 cell model expressing wild-type or mutant human SOD1 [142]. Subsequent studies found SOD1 secretion in association with purified EVs from different sources, including the finding that astrocytes expressing mutant SOD1 could induce selective death of motor neurons through EV transfer. Misfolded mutant SOD1 has also been shown to be able to transfer between NSC-34 cells via EVs, as well as between primary SOD1-overexpressing mouse spinal cord cells [143]. A recent study comparing levels of SOD1 and ALS-related biomolecules in plasma EVs between patients with ALS and healthy controls has provided support for further characterization of ALS-related SOD1 levels in various types of EVs and has implicated it as a biomarker for ALS [144]. Earlier studies have identified TDP-43 in CSF EVs from patients with ALS by western blotting and mass spectrometry [139]. TDP-43 has been found to be enriched in EVs in the conditioned media of neuroblastoma cells expressing TDP-43, as well as in EVs extracted from the CSF of patients with ALS and frontotemporal dementia [145, 146]. These results suggest that the transmission of TDP-43 is achieved by EV release. Moreover, a study published in 2015 using a luciferase fragment-TDP-43 fusion peptide showed that cells preferentially take up oligomer TDP-43 encapsulated in EVs, leading to greater toxicity. This study demonstrated the transfer of oligomer TDP-43 between neurons via EVs and the axonal transportation of TDP-43 after uptake from the medium [147]. Studies have also shown that the TDP-43 oligomers in EVs are more toxic than free TDP-43; thus, TDP-43 in EVs is considered a potential marker of ALS.

**Table 3:** The role of extracellular vesicles in amyotrophic lateral sclerosis

Source of EVs	Content with biomarker potential	Mouse and cell models for mechanistic studies	Downstream molecules	References
NSC-34 cells, astrocytes, and plasma	SOD1	NA	NA	[142–144]
CSF and HEK-293 cells	TDP-43	CamKIIa-hTDP43_NLSm_ mice	NA	[139, 146, 147]
SH-SY5Y and N2A cells	FUS	NA	DHX9, Matrin-3, DDX3X, and Caprin-1	[148]
NSC34 cells	DPR	NA	NA	[141]
CSF	miR-34a, miR-335, and miR-625-3p	Motor neurons	BCL2, IL6R, MAP3K7, PLCG1, PPARA, PRRC2B, CD47, CSNK2A1, HSPA8, and TRIAP1	[149]
CSF	miR-124-3p	SOD1G93A mice	NA	[150]
CSF and Serum	miR-132-5p, miR-132-3p, miR-143-3p, miR-143-5p, and miR-574-5p	NA	TDP-43	[151]
Serum	miR-27a-3p	NA	NA	[152]
Serum	miR-342-3p, miR-1254, miR-587, miR-298, miR-4443, and miR-450a-2-3p	iNeurons	NDST4	[153]
Astrocytes	miR-155, miR-21, and miR-146a	SOD1G93A mice	Apoptosis, kinesin-1, nNOS, and synaptic-related genes	[154]

*NA* No accessible data in the study; *CSF* Cerebrospinal fluid; *iNeurons* Induced pluripotent stem cell (iPSC)-derived neurons; *NSC-34 cells* Mouse motor neuron-like hybrid cell line; *HEK-293 cells* Human embryonic kidney 293 cells

In addition to SOD1 and TDP-43, other ALS-related targets are also contained in secreted EVs, albeit with a lower concentration. ALS-related mutations in *FUS* can lead to varying degrees of mislocalization of FUS in the cytoplasm, possibly related to the formation of stress granulosa structures. A previous study in familial ALS demonstrated that FUS interacts with RNA-binding proteins Matrin-3 and hnRNPA1 to form mutant complexes. This study also confirmed the presence of FUS in EVs, providing evidence for the spread of FUS between pathological cells, which may allow for the diagnosis of ALS [148]. GGGGCC repeats in *C9orf72* are the most common cause of ALS and the basis of ALS vesicle trafficking [155, 156]. RNA containing the GGGGCC repeat sequences is translated into DPRs which can form aggregates in the CNS of patients with ALS [157]. Intercellular diffusion of DPR can occur through EVs, and DPR-containing EVs have been isolated from ALS spinal motor neurons containing C9orf72 repeat expansions, suggesting biomarker opportunity for ALS [141]. The presence of other ALS-related protein mutants in EVs, including valin-containing protein [158], sequestosome 1 [159], and Tank-binding kinase 1 [160], may also be used to diagnose ALS.

Apart from proteins, EV miRNAs have become promising tools for better diagnosis of ALS because some miRNAs may alter the expression of proteins involved in ALS. Rizzuti et al*.* [149] found that miR-34a, miR-335 and miR-625-3p in CSF EVs may be used as biomarkers for ALS. Yelick et al*.* [150] provided preliminary evidence supporting the use of miR-124-3p in CSF EVs as an indicator for ALS disease staging. Freischmidt et al*.* [151] measured miRNA levels in EVs from the CSF and serum of 22 patients with sporadic ALS and 24 healthy controls. In patients with ALS, EV-encapsulated miR-132-5p, miR-132-3p and miR-143-3p were significantly reduced, while miR-143-5p and miR-574-5p were significantly increased, implicating their biomarker potential for ALS diagnosis. Xu et al*.* [152] indicated that miR-27a-3p in serum EVs was significantly reduced in patients with ALS, suggesting that it may be a potential diagnostic biomarker for ALS. Lo et al*.* [153] demonstrated that miR-342-3p, miR-1254, miR-587, miR-298, miR-4443 and miR-450a-2-3p in serum and brain-tissue EVs reflect the state of CNS disease in ALS, thus providing an opportunity for possible diagnosis. Gomes et al*.* [154] found that the inflammatory-associated miRNAs miR-155, miR-21, and miR-146a are depleted in EVs both originating from the spinal and from cortical astrocytes in ALS mice, and may be used as biomarkers for ALS.

In conclusion, EVs have potential applications in the pathological investigation, early (possibly pre-clinical) diagnosis, and treatment management of ALS. They play a role in disease pathogenesis through the transfer and subsequent intracellular accumulation of pathological proteins such as TDP-43, SOD1, and FUS. Studies have reported dysregulation of protein and microRNA cargos of EVs in cell and animal models of ALS and in patients. However, there are multiple difficulties in developing EVs as biomarkers. The different biofluids (CSF, plasma, and serum) used for investigation and the different methods for isolating EVs are among the multiple reasons for the lack of consensus among studies.

### EVs and HD

HD is clinically characterized by progressive motor deficits (e.g., chorea, oculomotor abnormalities, verbal ataxia, and dysphagia), cognitive dysfunction (dementia) and psychiatric disorders (e.g., depression, anxiety, and apathy), and pathologically by the loss of long-projection neurons in the cortex and striatum [161]. Progressive motor failure is a major cause of complications, leading to death within 15 to 20 years of onset. HD displays autosomal dominant inheritance caused by CAG (cytosine-adenine-guanine) repeats (≥ 36) of the Huntington’s disease chorea gene (IT15) on chromosome 4, resulting in an abnormal number of N-terminal glutamine repeats (polyQ) in mutated huntingtin protein (mHTT) [162].

EVs can cross the BBB and cause aggregation of mHTT in HD, resulting in mitochondrial dysfunction and cell death (Table 4). In HD, the aggregation of mHTT in neurons and the spread of mHTT aggregates between cells were  revealed by the internalization of synthetic peptide (44Q) into cells and the formation of cytoplasmic aggregates in vitro and in animal experiments [163–165]. Interestingly, in three patients with HD, mHTT aggregates were found in the allografts of striatal tissue, confirming the spread mHTT into genetically unrelated tissue [166]. EVs may be involved in the proliferation of mHTT protein by delivering proteins or nucleic acids, suggesting its potential as a diagnostic marker for HD. In a previous study, EVs released from the fibroblasts of patients with HD were injected into the ventricles of neonatal mice, and they led to HD-related pathology and behaviors [167]. In an HD model, Lee et al*.* [168] demonstrated that the adipose-derived stem cell (ASC)-derived EVs alleviated disease progression by reducing mHTT aggregation and apoptotic protein levels. Furthermore, immunocytochemistry and western blot confirmed that the ASC-derived EVs could release neurotrophic factors and significantly reduce mHTT aggregation, mitochondrial dysfunction, and apoptosis in neuronal cells. The density of mHTT aggregates has been shown to decrease following injection of astrocyte-derived EVs into the striatum of HD 140Q knock-in mice. Interestingly, the mHTT protein was not detected in the EVs secreted by the primary astrocytes, suggesting that the astrocyte-derived EVs may be used for treating HD [169]. The neuroprotective synaptic chaperone cysteine string protein α mediates the cellular export of polyglutamine expanded protein 72Q HTT^exon 1^ via EVs, again showing potential value for HD therapy [170].

**Table 4:** The role of extracellular vesicles in Huntington’s disease

Source of EVs	Content with biomarker potential	Mouse and cell models for mechanistic studies	Downstream pathway or molecule	References
SH-SY5Y cells	mHTT	NA	NA	[166]
CAD cells	CSPα	CSPα knock-out mice	SOD-1	[170]
HEK-293 cells	miR-124	R6/2 HD mice	RE1-Silencing Transcription Factor	[171]

*NA* No accessible data in the study; *HEK-293 cells* Human embryonic kidney 293 cells; *CSPα* cysteine string protein α

The miRNA content in EVs has not been studied in HD, and the use of EV miRNAs as biomarkers for HD diagnosis has not been reported. Several studies have reported a decreased level of miR-124 in the brains of patients with HD. However, a study reported that treatment of R6/2 transgenic HD mice by EV-mediated delivery of miR-124 [171] did not improve motor symptoms. Nevertheless, the function of miR-124 in EVs and its possible association with HD deserve further investigation.

### Therapeutic potential of EVs for neurodegenerative diseases

Various studies have suggested that EVs have several advantages over conventional synthetic carriers, such as their ability to cross the BBB and the low tendency to evoke an immune response, opening a new frontier for their usage in drug delivery and as therapeutics for neurodegenerative diseases. Research on EVs as a therapeutic vector is increasing. There have been at least dozens of phase 1/2 clinical trials registered for cancer, SARS-CoV-2 and AD, and treatment methods include stem cell-derived EVs, autologous EVs or drug-loaded EVs [172]. Some studies have confirmed that exosomes can be used as a promising drug delivery platform for target therapies against PD and other neurodegenerative diseases [173]. Therefore, cell-derived EV-based carrier systems have attracted considerable interest [174]. EVs have been used in murine models of PD and AD to reduce pathological protein accumulation. EVs containing *BACE1* siRNAs have been used in C57BL/6 mice, resulting in an overall 60% reduction of *BACE1* mRNA and 55% decrease of Aβ1-42 level [175]. In another study, EVs with α-syn siRNA were peripherally injected into S129D α-syn transgenic mice, which decreased the level of α-syn aggregates in brain regions pathologically affected in PD [176]. Bonafede et al. showed that exosomes derived from murine adipose-derived stromal cells are able to protect NSC-34 cells (which overexpress human SOD1 mutants) from oxidative damage [177], and similar results were reported by Lee et al. [178]. In R6/2 mouse-derived neuronal cell model of HD, EVs derived from ASCs slowed the progression of the disease and reduced the levels of mHTT aggregates and apoptotic proteins, showing the potential to treat HD [168]. Better therapeutic efficacy can also be achieved by modifying EVs. Research by Didiot et al. [179] showed that EVs loaded with hydrophobic siRNAs targeting HTT mRNA were efficiently internalized by mouse primary cortical neurons and promoted dose-dependent silencing of HTT mRNA and protein. Thus, both natural exosomes and modified EVs could play important roles in the treatment of HD.

The results of these studies suggest that EVs have great potential as a novel therapy for the treatment of neurodegenerative diseases. EVs can penetrate the BBB in a bidirectional manner, providing a means of communication to and from the CNS [180]. They are stable in the peripheral circulation and able to protect their cargos from degradation [181]. However, the wide variety of EV sources and isolation methods have limited the reproducibility and comparability across studies. There are many methods for EV isolation, each with distinct advantages and disadvantages. Therefore, it is crucial to follow the International Society for Extracellular Vesicles guideline for EV characterization to maximize the effectiveness and enable more reliable comparisons between studies. Further explorations in the clinical context are also needed. Moreover, while EVs are found in human CSF, urine and blood, it is unclear which source of EVs is better for treatment [182]. Therefore, when using EVs as a treatment plan, a full understanding of their modes of action in the disease and appropriate design of EVs are essential for improving the therapeutic effects (Table 5).

**Table 5:** Use of extracellular vesicles (EVs) to treat neurodegenerative diseases

Disease	Source of EVs	Mouse and cell models for mechanistic studies	Results	References
AD	MSCs	5 × FAD mice	Reduce chronic inflammation, facilitate the Aβ clearance	[183]
	MSCs	C57BL/6 mice	Promote neurogenesis and cognitive function recovery	[184]
	hucMSC	APP/PS1 mice	Repair cognitive disfunctions, clear Aβ deposition	[185]
	BM-MSCs	APP/PS1 mice	Reduce the Aβ plaque burden and the amount of dystrophic neurites in both the cortex and hippocampus	[186]
	BM-MSCs	APP/PS1 mice	Increase the expression of microRNA-146a in the hippocampus, decrease the levels of nuclear factor kappa B (NF-κB) in astrocytes, leading to synaptogenesis and the correction of cognitive impairment	[91]
	MSCs	APP/PS1 mice	Suppress the inducible nitric oxide synthase (iNOS) in cultured primary neurons and ameliorate the neural impairment of CA1 synaptic transmission in an AD mouse model	[187]
	MSCs	APP/PS1 mice	Improve learning and memory capabilities with reduced plaque deposition and Aβ levels and normalize levels of inflammatory cytokines	[188]
	Neuroblastoma	APP transgenic mice	Decrease Aβ levels, amyloid deposition, and Aβ-mediated synaptotoxicity in the hippocampus	[189]
	Neuronal	APP transgenic mice	Decrease Aβ and amyloid deposition	[190]
	Human adipose tissue-derived mesenchymal stem cells	N2a cells	Decrease the levels of Aβ	[191]
	Dendritic cells	C57BL/6 mice	Decrease BACE1 and Aβ	[175]
PD	Macrophages	PD mouse	Reduce brain inflammation	[192]
	Dendritic cells	S129D α-Syn transgenic mice	Reduce α-syn and intraneural protein aggregation	[176]
	Dental pulp stem cells	ReNcell VM immortalized human neural stem cell	Reduce the production of ROS and consequently apoptosis	[193]
	hucMSCs	SH-SY5Y cell	Reduce the dopaminergic neuron loss and apoptosis and upregulate the levels of dopamine in the striatum	[194]
	Dendritic cells	C57BL/6 male mice	Clear pre-existing extracellular Aβ	[175]
ALS	Murine adipose-derived stromal cells	NSC-34 cells	Increase ALS motoneuron survival, probably counteracting the apoptosis pathway	[177]
	Adipose-derived stem cell	G93A ALS mice model neuronal cells	Reduce mutant SOD1 aggregation and restore mitochondrial protein function	[178]
HD	Adipose-derived stem cells	HD model	Reduce huntingtin protein aggregation and apoptotic protein levels, reduce mutant huntingtin (mHtt) accumulation in neuronal cells	[168]
	U87 glioblastoma cells	Wild-type FVBNj mice	Promote dose-dependent silencing of HTT and protein	[179]

## Conclusions and prospects

To sum up, EVs are involved in the development and progression of neurodegenerative diseases. EVs in the microenvironment carry and transmit oxidative and inflammatory signals (such as proteins and miRNAs) secreted by neurons and glial cells. EVs may also directly transfer pathogenic substances (such as protein aggregates) from one cell to another. Therefore, the physiology and pathology of brain cells and even the progression of neurodegenerative diseases such as AD, PD, ALS and HD may be affected by EVs [195]. In this review, we discuss current advances on the roles of EVs in the development of neurodegenerative diseases, as well as their biomarker and therapeutic potentials in neurodegenerative diseases. EVs have also been found to be involved in neuronal self-rescue, where neurons remove harmful substances by secreting EVs; this could promote or inhibit disease depending on their content and the intrinsic nature of the disease. However, whether neurons preserve or pass proteins to other neurons via EVs, leading to more serious consequences, needs to be further explored.

EVs have been used as experimental tools for the diagnosis and treatment of animals [96]. Importantly, EVs in blood, CSF, urine and saliva contain various biomarkers, thus being a non-invasive tool for the early detection of disease and development of treatment strategies. Moreover, an increasing number of clinical trial have investigated the clinical applications of EVs. For example, a clinical study at University of Alabama at Birmingham aimed to determine biomarkers for PD susceptibility and/or progression from exosome-proteomes derived from PD patients (ClinicalTrials.gov Identifier: NCT01860118). Another study at Ruijin Hospital, Shanghai, China evaluated the safety and efficacy of exosomes derived from allogenic adipose mesenchymal stem cells in subjects with AD (ClinicalTrials.gov Identifier: NCT04388982). Molecular biomarkers, such as EV miRNAs, may provide new insights into the diagnosis and treatment of neurodegenerative diseases such as AD, PD, ALS, and HD. As evidenced by the studies covered in this review, with the development of basic research, EVs have shown great potential in neurodegenerative disease research, especially as a target-drug-carrier for the treatment of neurodegenerative diseases. In this review, we also present the recent advances in the analysis of EVs, which may lead to the discovery of new biomarkers for neurodegenerative diseases and facilitate the identification of new therapeutic targets. It has recently been suggested that altering the release level of EVs may be beneficial for therapeutic approaches in some neurodegenerative diseases, particularly at the onset of the disease. The current dilemma is that there are no published clinical trials on the role of EVs in treating neurodegenerative disorders. Indeed, previous research on EVs has focused on the effectiveness, but which components are responsible for the observed efficacy has not been established yet; this has raised doubts on the safety and effectiveness of EVs. Finally, there remains a lack of strict standards for the quality management of EVs. Different tissue sources, donor cells and preparation methods may result in heterogeneous EVs. In addition, due to the different in vivo and in vitro models among laboratories, the effective concentrations of EVs and intervention methods in different diseases have not been finalized, thus hindering their clinical translation. As such, it is crucial to develop more efficient separation methods to deal with these issues. High-quality cohort design and development of high-tech hardware equipment and artificial intelligence can assist biomarker discovery/validation/clinical translation. The correlation between multi-omics data and imaging biomarkers is the future direction of research.

Some issues need to be addressed before using EVs for treatment of neurodegenerative diseases. For example, the precise content sorting and regulatory mechanisms of secreted EVs remain largely unknown. Additionally, advanced selection and isolation techniques are required to better distinguish EVs from other extracellular particles. Most importantly, more studies are needed to improve the performance of EV-carrying biomarkers for clinical diagnosis. Further studies are also needed to examine the relationship between abnormal upregulation or downregulation of EV biomarkers and disease progression. Despite these obstacles, the use of EVs as a potential biomarker and a treatment for neurodegenerative diseases is attractive and worthy of future research.

## Data Availability

Not applicable.

## Abbreviations

AD: Alzheimer’s disease
PD: Parkinson’s disease
ALS: Amyotrophic lateral sclerosis
HD: Huntington’s disease
EVs: Extracellular vesicles
CNS: Central nervous system
CSF: Cerebrospinal fluid
BBB: Blood–brain barrier
NSC: Neural stem cell
sEV: Small extracellular vesicle
MV: Microvesicle
ESCRT: Endosomal sorting complex required for transport
Alix: Apoptosis-linked gene 2-interacting protein X
TSG101: Tumor susceptibility gene 101
MVB: Multivesicular body
ILVs: Intraluminal vesicles
LSE: Late sorting endosome
ESE: Early sorting endosome
MHC: Major histocompatibility complex
L1CAM: L1 cell adhesion molecule
Aβ: Amyloid β
α-syn: α-Synuclein
APP: Amyloid precursor protein
t-tau: Total tau
p-tau: Hyperphosphorylated tau
SCD: Subjective cognitive decline
AUC: Area under the curve
SOD: Cu/zinc superoxide dismutase
TDP-43: Tar DNA binding protein-43
FUS: Fused in sarcoma
DPR: Dipeptide repeating protein
hnRNP: Heterogeneous nuclear ribonucleoprotein
mHTT: Mutated huntingtin protein
CSPα: Cysteine string protein α
